# Genome-wide identification and functional characterization of the PheE2F/DP gene family in Moso bamboo

**DOI:** 10.1186/s12870-021-02924-8

**Published:** 2021-03-29

**Authors:** Long Li, Qianqian Shi, Zhouqi Li, Jian Gao

**Affiliations:** 1 College of Forestry, Northwest A&F University, Yangling, 712100 Shaanxi China; 2 College of Landscape Architecture and Art, Northwest A&F University, Yangling, 712100 Shaanxi China; 3 International Center for Bamboo and Rattan, Key Laboratory of Bamboo and Rattan Science and Technology, State Forestry Administration, Beijing, China

**Keywords:** E2F/DPs, Moso bamboo, Expression, Cell cycle, Bamboo shoot

## Abstract

**Background:**

E2F/DP proteins have been shown to regulate genes implicated in cell cycle control and DNA repair. However, to date, research into the potential role of the Moso bamboo E2F/DP family has been limited.

**Results:**

Here, we identified 23 E2F/DPs in the Moso bamboo genome, including nine E2F genes, six DP genes, eight DEL genes and one gene with a partial E2F domain. An estimation of the divergence time of the paralogous gene pairs suggested that the E2F/DP family expansion primarily occurred through a whole-genome duplication event. A regulatory element and coexpression network analysis indicated that *E2F/DP* regulated the expression of cell cycle-related genes. A yeast two-hybrid assay and expression analysis based on transcriptome data and in situ hybridization indicated that the PheE2F-PheDP complex played important roles in winter Moso bamboo shoot growth. The qRT-PCR results showed that the PheE2F/DPs exhibited diverse expression patterns in response to drought and salt treatment and diurnal cycles.

**Conclusion:**

Our findings provide novel insights into the Moso bamboo E2F/DP family and partial experimental evidence for further functional verification of the PheE2F/DPs.

**Supplementary Information:**

The online version contains supplementary material available at 10.1186/s12870-021-02924-8.

## Background

Moso bamboo (*Phyllostachys edulis*) generates nearly 5 billion US dollars annually in economic value. It is the most important non-timber product used for commercial purposes in East Asia due to its fast growth rate [1, 2]. During Moso bamboo shoot growth, the height increase was created by simultaneous cell division and cell elongation. Cell division played critical roles during the winter and early growth periods, while cell elongation was predominant during the late growth period.

The E2F/DP transcription factors in higher plants are categorized into E2F, DP, and DEL (DP-E2F-like) groups based on their conserved domains [3]. The E2F group gene contains four functional domains including an RBR-binding domain, a ‘marked box’ domain, a DNA-binding domain, and a leucine zipper dimerization domain, while the members of the DP group lack the RBR-binding domain, a ‘marked box’ domain compared with E2F. The DEL group genes are considered to be atypical E2F/DPs and act in monomeric form, and the DEL genes only contain a duplicated DNA-binding domain [4].

The E2F/DP family has eight members in *Arabidopsis*. During cell proliferation, the canonical AtE2Fs reportedly play an antagonistic role, because E2Fc is a negative regulator, whereas E2Fa and E2Fb are positive regulators [5–7]. E2Fa and E2Fb interact with DPa to activate the cell cycle and cell proliferation-associated gene expression through the organization of their leucine zipper dimerization domains [8].

The E2F member might perform distinct roles in controlling the cell fate determination [3]. In mammals, the E2F signaling pathway is essential for cell growth and cell proliferation [9]. Studies on E2F in *Arabidopsis* have indicated that plants also have all the core regulators in the E2F signaling pathway, such as cyclin-dependent kinase inhibitors (CKIs), retinoblastoma (RBs), cyclins, and cyclin-dependent kinases (CDKs). In plants, E2F/DP binds to the E2FAT (TTTCCCGCC) motif in its target genes, playing a role in promoting transcription [10].

Previous studies identified 12 PhE2F/DP transcription factors from the first version of the Moso bamboo genome database [11]. However, because of limits in the draft genome sequence, a considerable number of *PhE2F/DPs* were still missing annotations. In addition, the expression changes and potential roles of the *PheE2F/DP* genes during bamboo shoot growth and in response to stress stimuli and diurnal cycles were still absent. In this study, a total of 23 E2F/DPs were identified based on the new version of the Moso bamboo genome annotation project, which provided chromosome-level de novo genome assembly. The phylogenetic relationships, gene structures, and conserved motifs of 23 *PheE2F/DP* genes were analyzed. The promoter analysis indicated that various *cis*-acting elements, including light response and hormone signaling as well as many transcription factor binding sites, were present in the promoter region of the *PheE2F* genes. We surveyed potential gene promoters containing E2F/DP binding sites within the Moso bamboo genome and identified 580 E2F/DP target genes. Based on bioinformatic predictions, we further studied the expression dynamics of the *PheE2F* genes during bamboo shoot growth and in response to stress stimuli and diurnal cycles. A yeast two-hybrid assay and an expression analysis were conducted to investigate the potential roles of PheE2F/DPs involved in Moso bamboo shoot growth. Our study provided new insights and some valuable information for the further functional verification of E2F/DPs in Moso bamboo.

## Results

### Identification and classification of the PheE2F/DP gene family

A total of 24 potential PheE2F/DP genes were identified in the Moso bamboo genome, including a PH02Gene25981.t1 with an incomplete E2F domain. To find whether the incomplete E2F domain in PH02Gene25981.t1 was caused by incorrect gene structure annotation in the bamboo genome or PH02Gene25981.t1 was merely just a pseudogene, we used three different methods to provide bioinformatic and experimental evidence. First, we used the CD-Search program from the NCBI to identified conserved domains involved in the whole *PH02Gene25981.t1* sequence, including exons, introns, 5′-UTRs and 3′-UTRs, but no complete E2F_DD or DP domains were identified in the *PH02Gene25981.t1* sequence. Second, we searched the expression information and potential splicing variants of *PH02Gene25981.t1* in many previously published and unpublished transcriptome sequencing data, including single molecule sequencing data [2, 12]; however, no valuable information was identified. Lastly, we designed different primer pairs for PH02Gene25981.t1 CDS and the exon that contained the partial E2F domain. Different RNA samples were isolated from different tissues, including the leaves, culms, rhizome, bamboo shoots, seeds, roots, flowers, and seedlings under various abiotic stresses, which were used as clone templates, but we did not obtain the CDS sequence of *PH02Gene25981.t1* from any of the tissues. Thus, we speculated that PH02Gene25981.t1 is just a pseudogene.

**Fig. 1:**
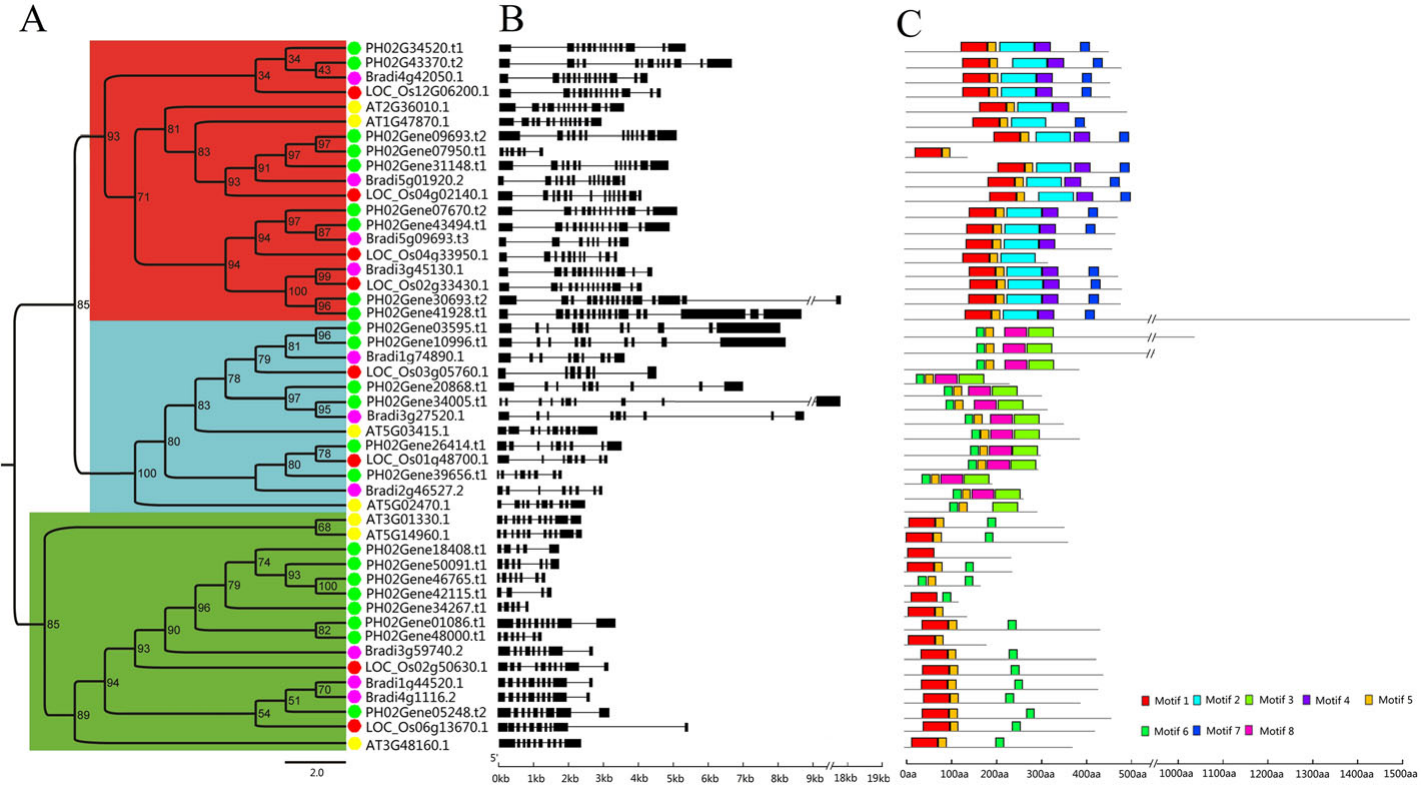
Phylogenetic tree, gene structure and conserved motif compositions of the PheE2F/DP families. **a** Phylogenetic tree relationship of the E2F/DP family. The *A. thaliana*, *O. sativa*, *B. distachyon*, and Moso bamboo proteins are marked with yellow, red, purple, and green dots, respectively. **b** Gene structures of the *E2F/DP* genes. Black boxes and black lines represent exons and introns, respectively. Scale bar: 1.0 kb. The −//− represents 8.0 kb. **c** Distribution of conserved motifs within each group. The scale bar represents 100 amino acids

To determine the evolutionary relationship of E2F/DPs in Moso bamboo and other model plants, a maximum likelihood tree of E2F/DP proteins from Moso bamboo, *Oryza sativa*, *Brachypodium* and *Arabidopsis* was constructed. For the 23 identified PheE2F/DP genes from Moso bamboo, the proteins could be classified into three functional groups: E2F, DP, and DEL [3]. Nine of these proteins were classified into the E2F group, six into the DP group and eight into the DEL group (Fig. 1a). In addition, a phylogenetic analysis of the E2F/DPs in four species indicated that PheE2F/DPs shared more sequence similarity with OsE2F/DPs and BdE2F/DPs than with AtE2F/DPs. For example, AT5G14960.1, AT3G01330.1, and AT3G486160.1 clustered together in the DEL group, showing a distant relationship to *Brachypodium*, rice, and Moso bamboo.

### Characterization of the PheE2F/DP gene family

A gene structural analysis of the E2F*/*DP family revealed that the genes within the same group shared similar intron lengths and numbers (Fig. 1b). The number of introns in the DP and DEL groups ranged from seven to nine and three to nine, respectively. The average numbers of introns in the E2F group were much higher than those in the other two groups, and the intron quantity ranged from five to 16.

Eight conserved motifs were identified in the E2F*/*DPs using the MEME online program (Fig. 1c and Additional file 1: Fig. S1). Motifs 1 and 6 represent the E2F_TD domain, and motifs 2 and 3 represent the E2F_DD and DP domains, respectively. Motifs 2, 4 and 7 were found exclusively in the E2F group, whereas motifs 3 and 8 appeared only in the DP group. Most DEL members shared motifs 1 and 5 with most E2F protein sequences. The motif analysis indicated that proteins involved in the same group shared several identical motifs outside the conserved domain mentioned above, indicating functional conservations within the same group.

**Fig. 2:**
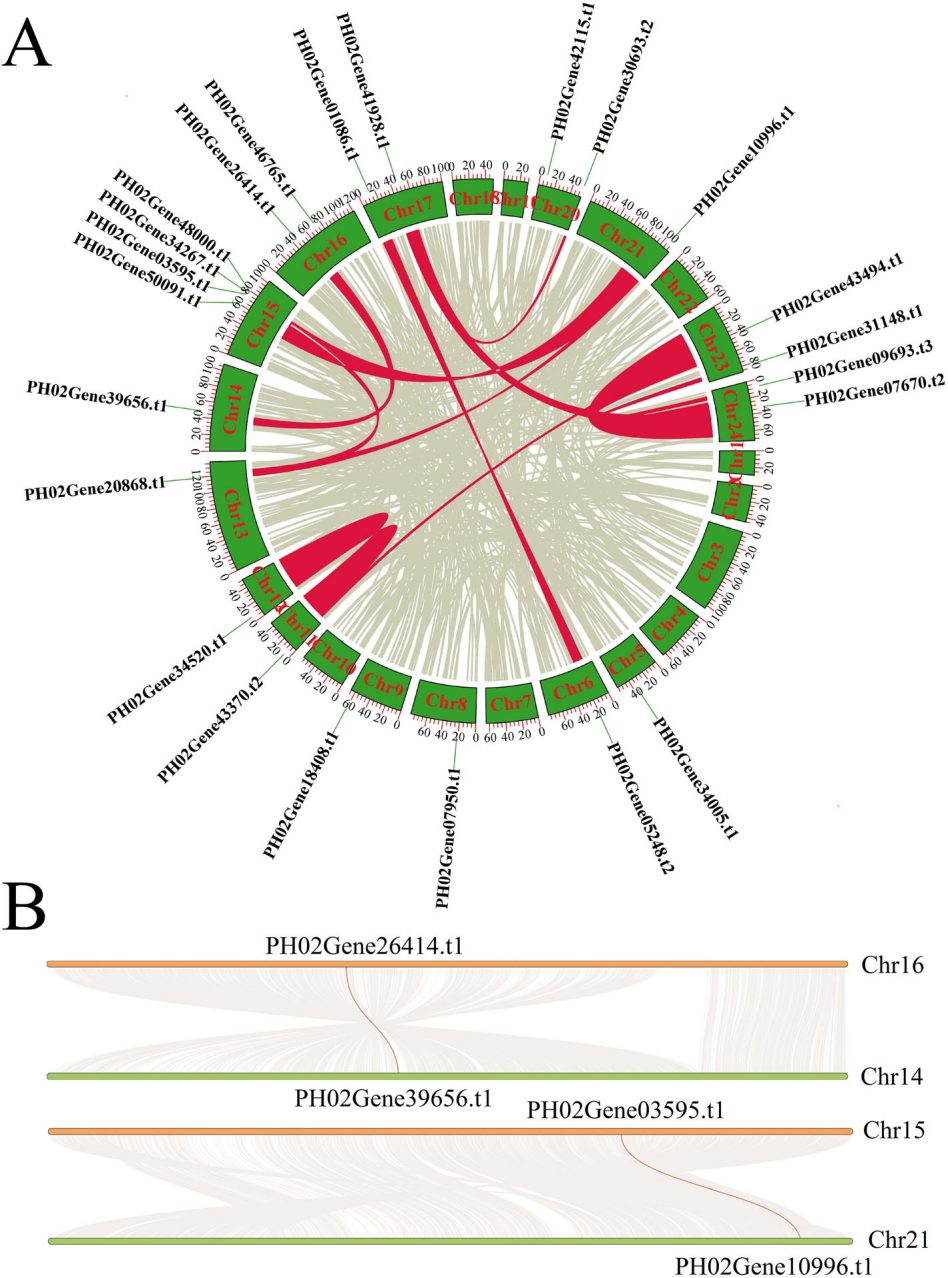
Gene location and collinearity analysis of the PheE2F gene family. **a** Syntenic relationships among synteny blocks carrying the 11 duplicated PheE2F/DP gene pairs. Red bars denote synteny blocks that harbored duplicated E2F/DP genes. Grey bars in the background indicate all the identified synteny blocks. **b** Example of collinearity analysis between two Moso bamboo chromosomes. Grey lines in the background indicate all the duplicated genes between two chromosomes, and the pairs of duplicated PheE2F/DP genes are connected by red lines

Gene duplication events play a critical role in the production of new functions and in gene expansion. Thus, we analyzed the potential duplication events of the PheE2F/DPs. No tandem duplication events were identified in the Moso bamboo E2F/DPs, but there were 16 genes pairing 12 E2F/DP paralogous pairs in synteny blocks in the Moso bamboo genome (Fig. 2a). For example, a large amount of highly conserved synteny blocks was observed between chromosomes (chr) 16 and 14, which contained Phe02Gene26414.t1 and Phe02Gene39656.t1, respectively (Fig. 2b)*.* Similar phenomena were observed between chr 15 and 21, which contained *Phe02Gene26414.t1* and *Phe02Gene39656.t1*, respectively, as well as other chromosome pairs that contained or did not contain paralogous E2F/DP pairs. Furthermore, we calculated the divergence time of the paralogous pairs (Additional file 1: Table S1). The divergence for most PheE2F/DP gene pairs (7 of 8) was approximately 6.5 to 13.5 mya, similar to the Moso bamboo whole-genome duplication event (7–12 mya) [13], and much later than those of *O. sativa*, *B. distachyon* and *A. thaliana.* These results suggested that a whole-genome duplication event played a critical role in the Moso bamboo gene expansion, including E2F/DPs.

### Regulatory network of PheE2F/DP in Moso bamboo

**Fig. 3:**
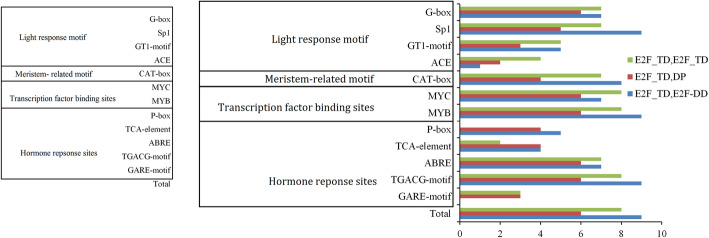
The quantity of *cis*-regulatory elements (CREs) in the *E2F/DP* promoters. Only identified elements with greater than five CREs were retained

The *cis*-elements, which are located in the promoter region, are essential to the spatial, temporal, and tissue-specific control of gene expression under external and internal environmental stimuli [14]. Thus, we screened the *cis*-element at the promoter regions (2000-bp upstream) of all the *PheE2F/DP* genes using the PlantCARE database. A large number of *cis*-elements including light response sites, hormone response sites, transcription factor binding sites and meristem-related motifs were found (Fig. 3). All 23 *PheE2F/DP* promoters contained at least one light response motif. MYB binding motifs were found in all 23 promoters of *PheE2F/DP* genes, and most *PheE2F/DP* promoters harbored MYC binding elements. Thirteen *PheE2F/DP* promoters contained P-box or GARE motifs that were responsive to gibberellin stimulation. The abscisic acid responsiveness (ABRE) site was abundant in the *PheE2F*/DP promoters, and this element was found in 21 promoters. In addition, the TGACG motif and TCA element, which are responsive to methyl jasmonate (MeJA) and salicylic acid stimulation, appeared in most of the *PheE2F/DP* promoter regions. Nineteen promoters contained CAT box, a meristem expression-related *cis*-element, indicating the importance of the *PheE2Fs* involved in cell division and proliferation. In addition, no significant difference was observed between different groups following a comparison of *cis*-acting elements.

**Fig. 4:**
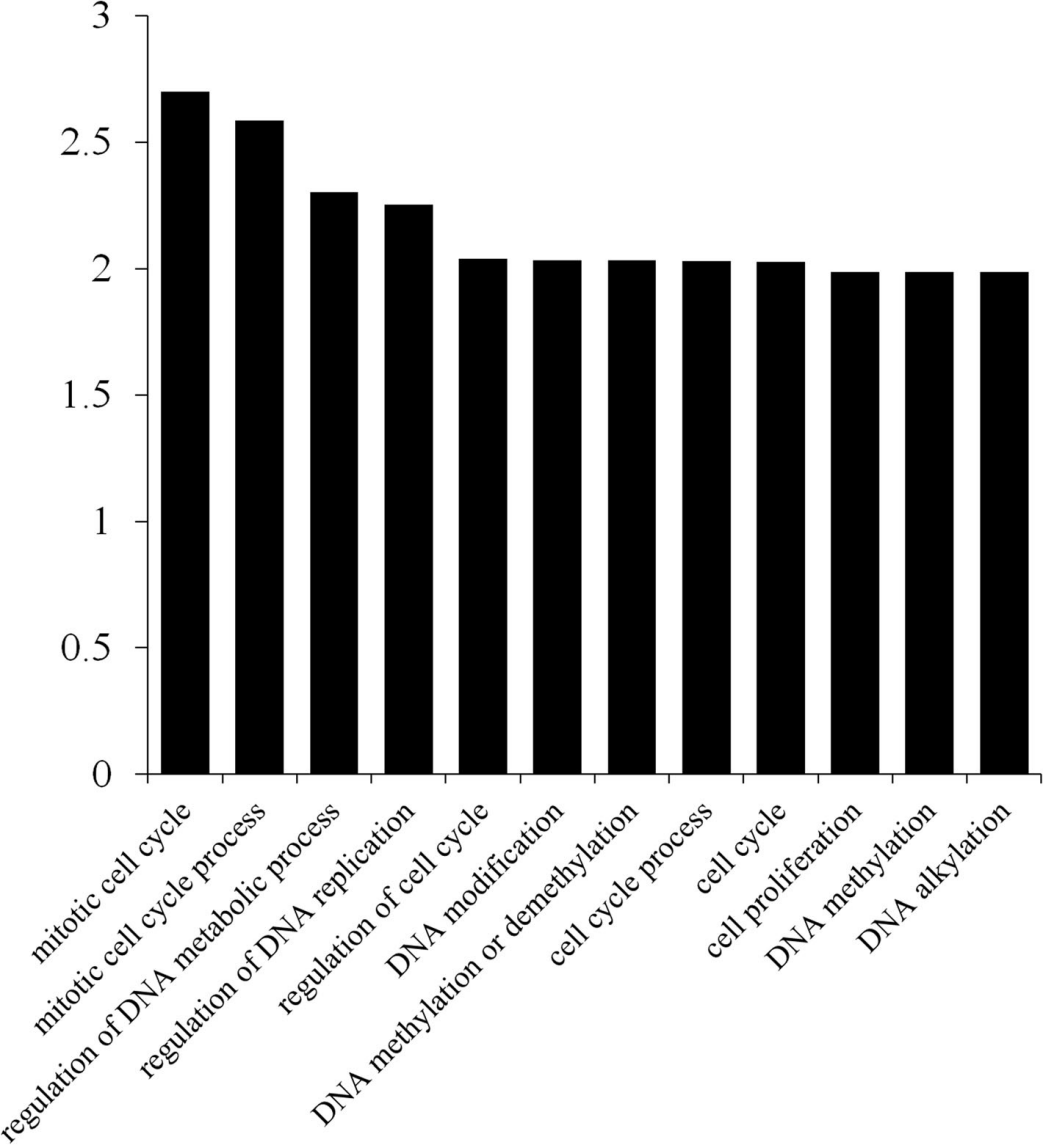
GO enrichment (biological processes) of PheE2F/DP-regulated genes (FDR < 0.05)

A previous study reported that E2F/DP binds to the E2FAT (TTTCCCGCC) motif of its target genes and regulates their transcription [10]. Thus, we screened the binding sites in the promoter regions of 51,074 protein-coding genes in the Moso bamboo genome using PlantCARE. Finally, 580 genes that contained E2F/DP binding sites in their promoter regions were identified. To explore the potential function of the genes regulated by PheE2F/DP, we performed a Gene Ontology (GO) enrichment analysis (Fig. 4). In the biological processes category, the groups with the highest abundance of genes included the mitotic cell cycle, mitotic cell cycle process, regulation of DNA metabolic process, regulation of DNA replication, and regulation of the cell cycle.

**Fig. 5:**
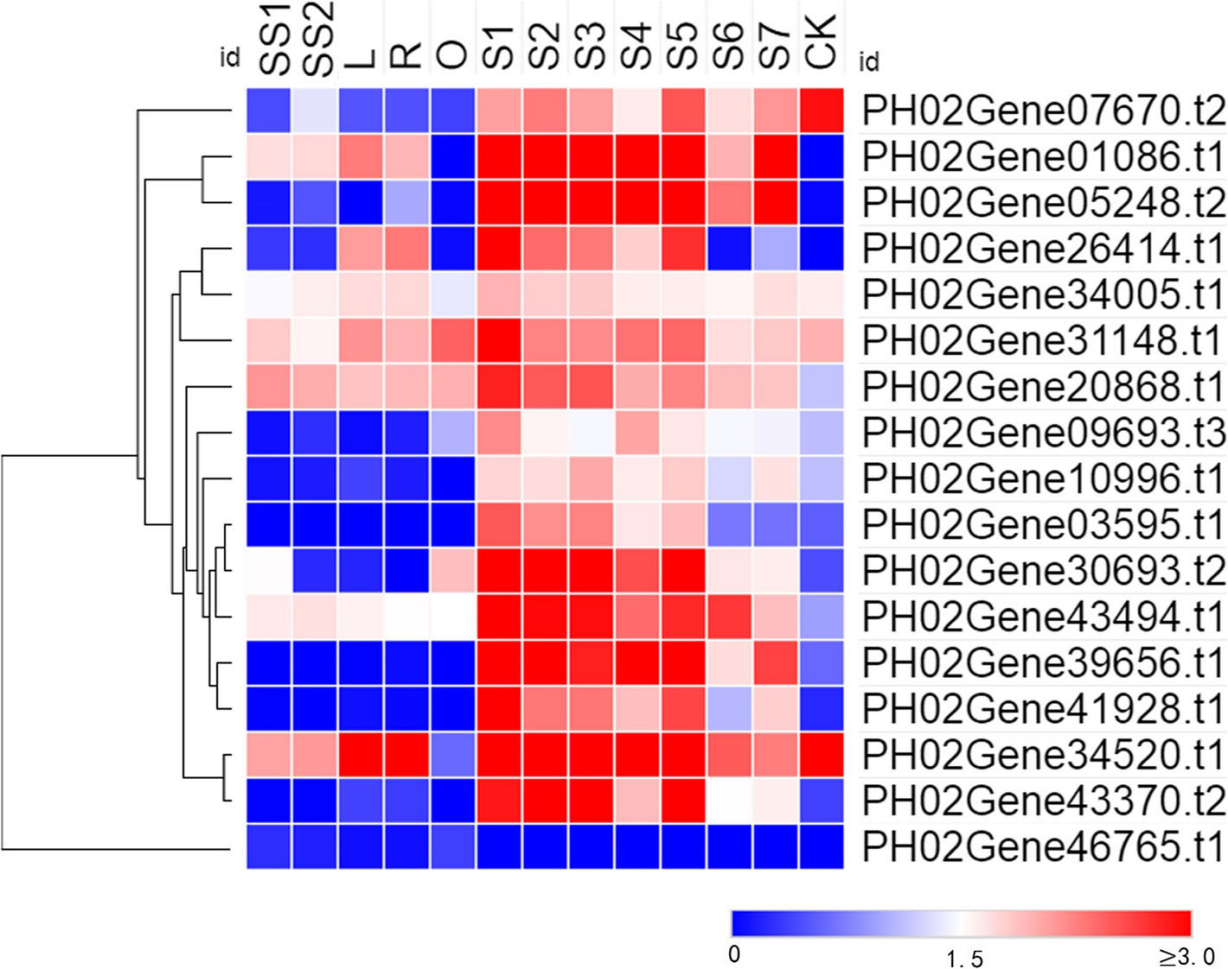
Expression patterns of *E2F/DP* genes in different Moso bamboo tissues. The color scale represents log2-transformed fragments per kilobase of transcript per million mapped reads (FPKM) values. Blocks with colors indicate high accumulation levels (red) or low accumulation levels (blue). S1-S7 and CK represent winter bamboo shoots; 50-, 100-, 300-, 600-, 900-, and 1200-cm height bamboo shoots; and one-year-old mature culms, respectively. SS1, SS2, L, R and O represent the 1.5-cm-tall seedling stem, 8-cm-tall height seedling stem, lateral bud, rhizome and outward rhizome, respectively

The transcriptome data generated from 13 different culm tissue samples were used to investigate the expression patterns of the *PheE2F/DPs.* Most E2F/DPs showed relatively high expression levels in winter bamboo shoots (S1) and spring bamboo shoots, especially during the early growth period (S2-S5) compared with the other growing culms, such as the rhizome (R) and seedling stems (SS1 and SS2). Of the 18 genes that were expressed in at least one tissue, 11 showed the highest expression level in winter bamboo shoots (S1) (Fig. 5). *PH02Gene34520.t1* and *PH02Gene43370.t2* were highly expressed in 50-cm-tall bamboo shoots (S2), while *PH02Gene46765.t1* exhibited the highest accumulation level in the outward rhizome (O). *PH02Gene05248.t2* and *PH02Gene10996.t1* were highly expressed in S4 (300-cm-tall bamboo shoots) and S5 (600-cm-tall bamboo shoots), respectively. The expression analysis results revealed that the *PheE2F/DPs* played important roles in the growth of the bamboo shoots, especially during the winter period.

**Fig. 6:**
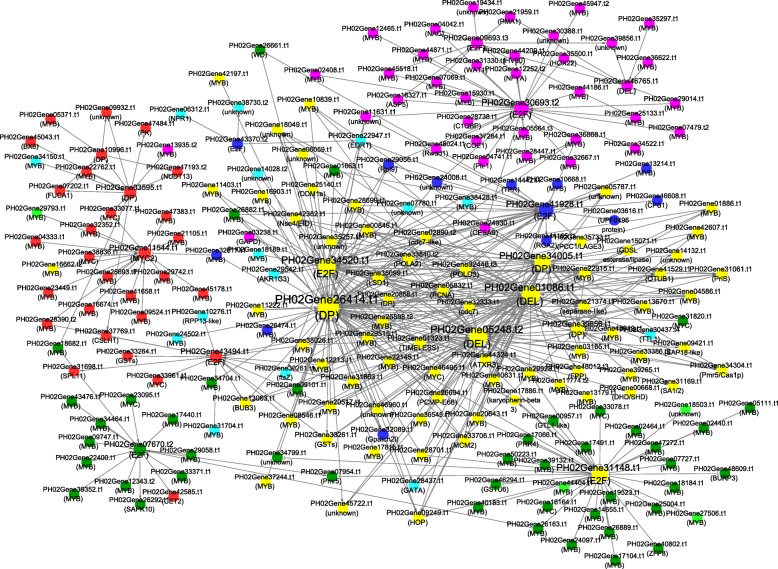
Graphic representation of a PheE2F/DP-centered interaction network. The graphic representation of the PheE2F/DP-centered interaction network shows the potential connections between the PheE2F/DPs and their predicted gene targets. The connections indicate the coexpression of genes with a Pearson correlation coefficient ≥ + 0.90 (arrows) or ≤ − 0.90 (T-type arrow). The larger circles in the network indicate genes with more connections. Yellow, red, purple light green, dark green, light blue and dark blue circles represent the genes with the highest expression levels in winter bamboo shoots, 0.5-m-tall bamboo shoots, outward rhizomes, 1.5-cm-tall seedling stems, 8-cm-tall seedling stems, lateral bud and rhizome, respectively

**Fig. 7:**
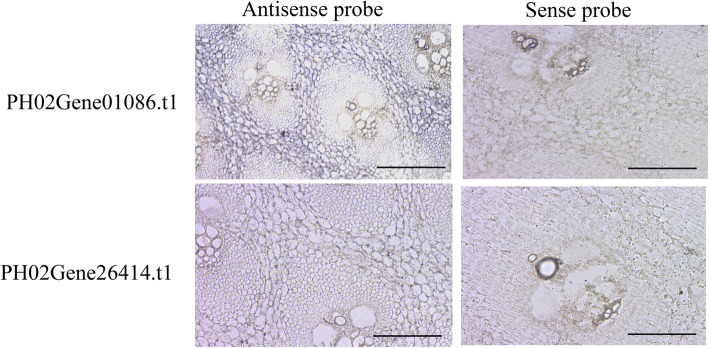
In situ hybridization of *PH02Gene01086.t1* and *PH02Gene20868.t1* in winter Moso bamboo shoots

To understand the regulatory network of PheE2F/DPs implicated in the different types of culm growth, we performed regulation tests between quantitative changes in the transcripts from the 13 different culm tissue samples. Based on the regulatory relationship obtained by predicting the regulatory elements in the promoter regions and PCC (R>0.90 or R<-0.90) screening, a total of 58 genes had a strong correlation with at least one PheE2F/DP gene (Fig. 6). In the coexpression network, the primary hub gene PH02Gene26414.t1, which had the highest connecting times, positively regulated the expression of *PH02Gene35099.t1* (*LSD1*), *PH02Gene44324.t1* (*ATXR3*), *PH02Gene09249.t1* (*HOP*), etc. In addition, PH02Gene05248.t2 and PH02Gene01086.t1 had the second and third-highest connecting times. All three hub genes reached the highest accumulation level in winter bamboo shoots, and all of them were regulated by PH02Gene08546.t1 (MYB), PH02Gene20517.t1 (MYB) and PH02Gene25898.t2 (MYB). In addition, all three genes regulated the expression of cell cycle-associated genes, including *PH02Gene32333.t1* (*CDKF*), *PH02Gene29974.t1* (*MRB1*), *PH02Gene36158.t1* (*SPR2*), and *PH02Gene33706.t1* (*MCM2*) as well as DNA replication-associated genes, including *PH02Gene32448.t3* (*POLD3*) and *PH02Gene37503.t2* (*POLA2*). Furthermore, many genes involved in RNA processing or environmental stress response showed high correlations with many E2F/DP members. The in situ hybridization data showed that the mRNAs of the hub genes *PH02Gene01086.t1* and *PH02Gene26414.t1* were highly expressed in ground tissues of winter bamboo shoots but showed low expression levels in vascular bundles (Fig. 7).

**Fig. 8:**
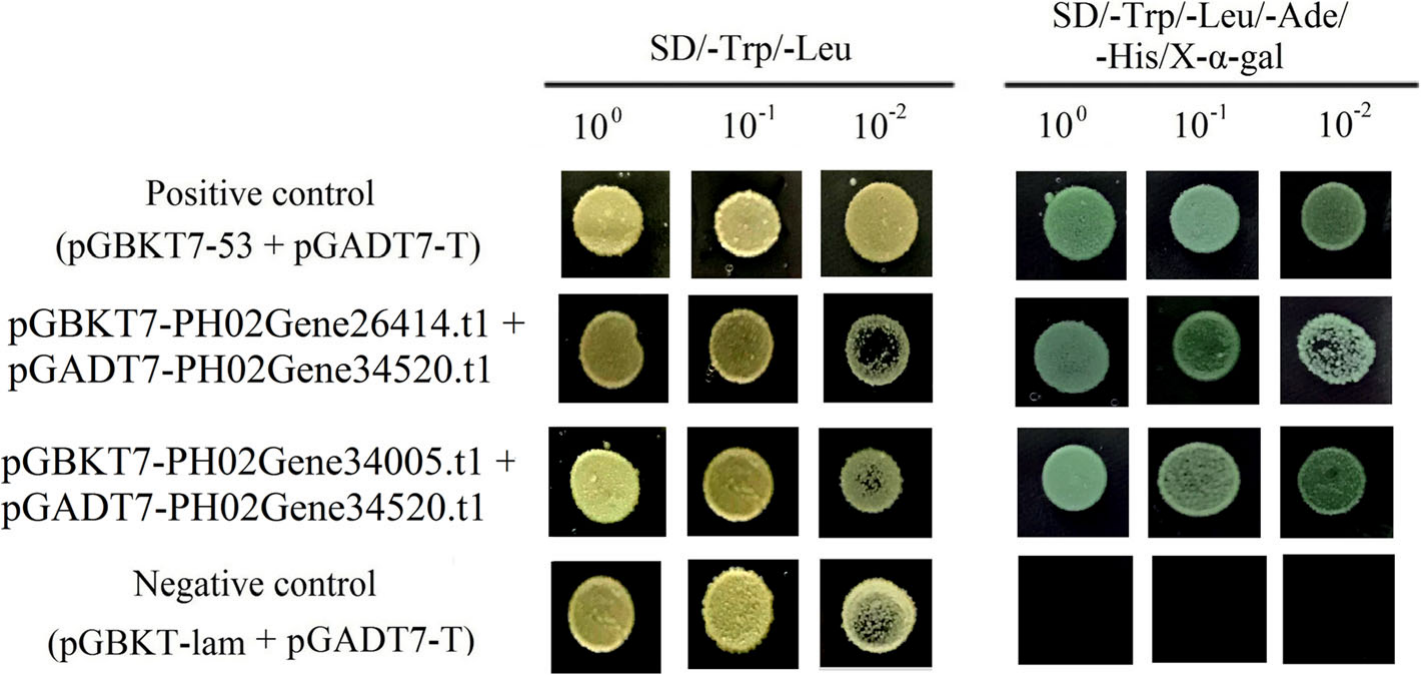
The interaction between PheE2Fs and PheDPs. Positive control, co-transformation with pGBKT7–53 and pGADT7-T; negative control, co-transformation with pGBKT7-Lam and pGADT7-T

In *Arabidopsis*, E2F can interact with DP to activate the cell cycle and cell proliferation-associated gene expression [8]. Thus, the yeast two-hybrid experiment was used to examine whether an E2F-DP complex also occurs in Moso bamboo. The three hub genes, *PH02Gene34520.t1* (*E2F*), *PH02Gene26414.t1* (*DP*), and *PH02Gene34005.t1* (*DP*), which showed the highest accumulation level in winter bamboo shoots, were selected and further tested. The combinations of pGBKT7-PH02Gene26414.t1 + pGADT7-PH02Gene34520.t1 and pGBKT7-PH02Gene34005.t1 + pGADT7-PH02Gene34520.t1 were co-transformed into yeast strain AH109. The Y2H results showed that the positive control, negative control, and both test transformants grew well on SD/−Leu/−Trp medium (Fig. 8). The positive control and the experimental group turned blue when the transformants had grown on SD/−Trp/−Leu/−Ade/−His/X-α-Gal media for 5 days. By contrast, the negative control could not grow on that nutritional selection media, which contained X-α-Gal. These experimental results indicated that PheE2Fs can interact with PheDPs in Moso bamboo.

### Expression patterns of *PheE2F/DPs* under abiotic stress treatment and diurnal rhythms

**Fig. 9:**
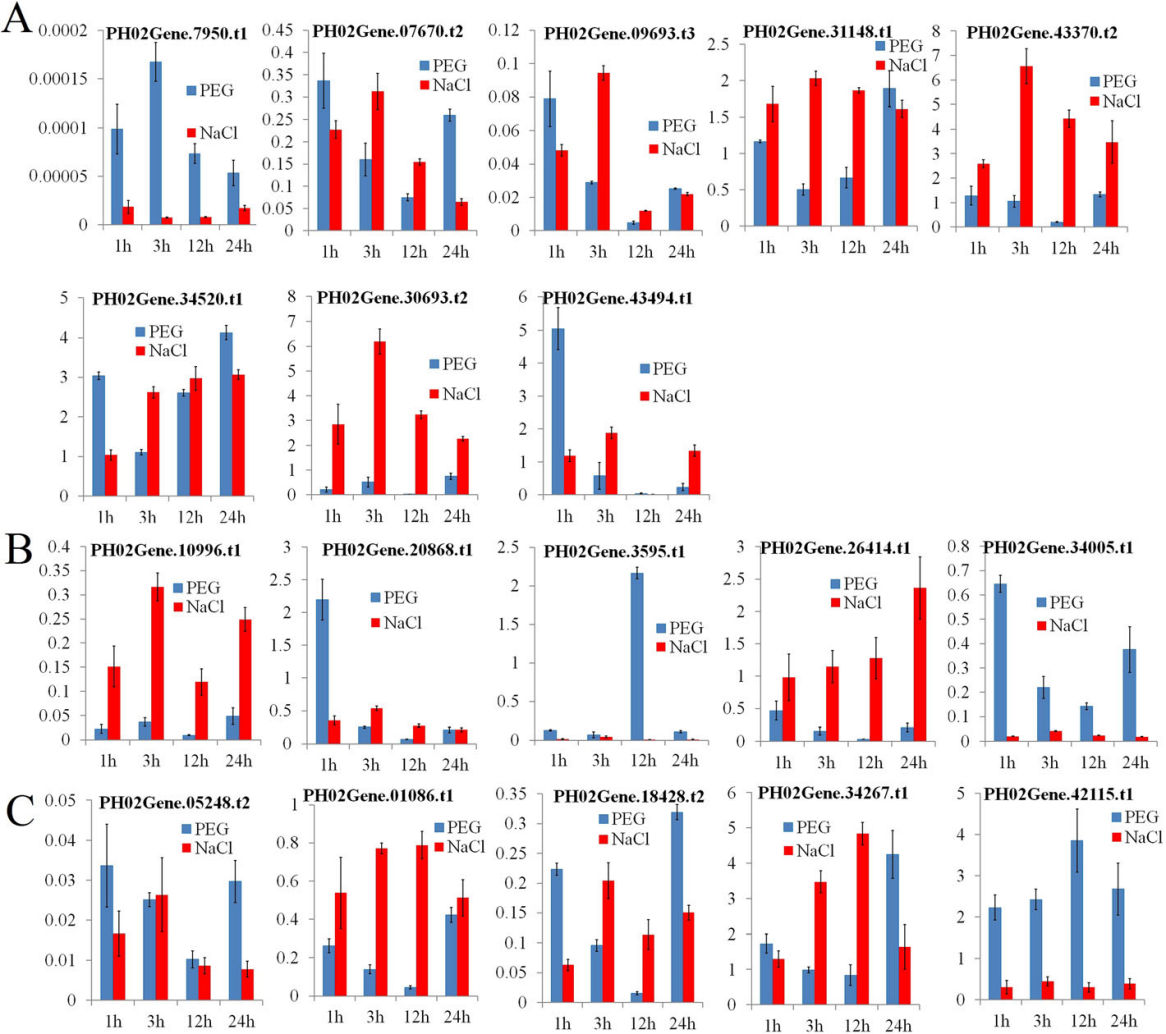
Expression patterns of PheE2F/DP genes after being treated for 1, 3, 12, and 24 h under drought stress (blue column) or salinity stress (red column). The Y-axis indicates the relative expression level. The 1, 3, 6, 12, and 24 labels (x-axis) indicate the treatment time (hours) under the corresponding abiotic stresses. The qRT-PCR data were normalized using the *TIP41* gene. For each time point, seedlings irrigated with tap water were regarded as a reference. **a** Relative expressions of E2F group genes under abiotic stresses. **b** Relative expressions of DP group genes under abiotic stresses. **c** Relative expressions of DEL group genes under abiotic stresses

The regulatory analysis revealed that most *PheE2F/DP* promoters harbored various types of hormones and light response elements. Hormone response elements, especially those of stress-related hormones such as abscisic acid, methyl limonate and salicylic acid, indicate that these genes might be induced or repressed by abiotic stress. To understand the expression changes of *PheE2F/DPs* under abiotic stress, the Moso bamboo seedlings were treated with drought (PEG) and salt treatments (Fig. 9). Under drought stress, the expression levels of *PH02Gene07950.t1*, *PH02Gene07670.t2*, *PH02Gene09693.t3*, *PH02Gene10996.t*1, *PH02Gene26414.t1*, *PH02Gene34005.t1*, *PH02Gene01086.t1*, *PH02Gene05248.t2*, and *PH02Gene18408.t2* were downregulated, while those of *PH02Gene34520.t1*, *PH02Gene34267.t1,* and *PH02Gene42115.t1* were upregulated. The expression of *PH02Gene03595.t*1, *PH02Gene20868.t1,* and *PH02Gene43494.t1* peaked at 12 h, 1 h, and 1 h, respectively. Under salt stress, *PH02Gene30693.t2*, *PH02Gene31148.t1*, *PH02Gene34520.t1*, *PH02Gene43370.t2, PH02Gene34267.t1*, *PH02Gene26414.t1*, and *PH02Gene34267.t1* showed upregulated expression trends, and the other *PheE2F/DPs* showed no significant differences in expression or downregulated expression trends.

**Fig. 10:**
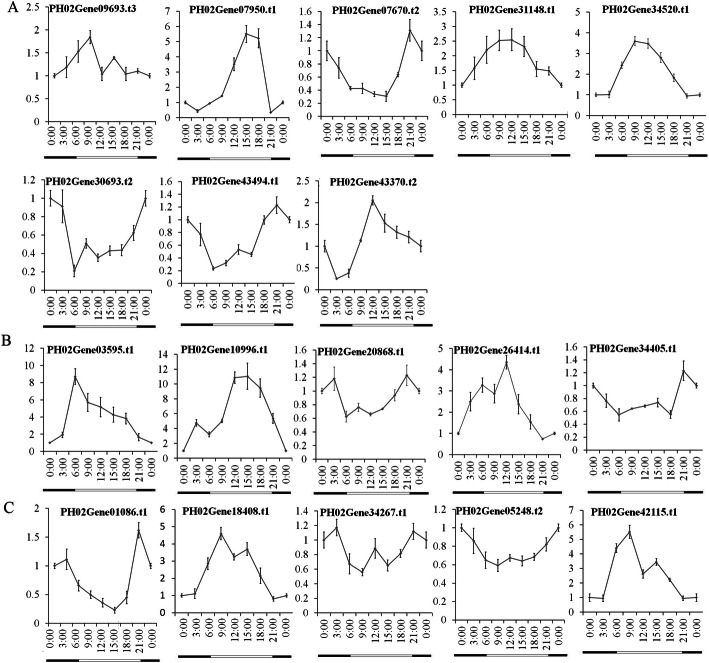
Diurnal changes in the relative transcript abundance of PheE2F/DP genes analyzed by qRT-PCR. The Y-axis indicates the relative expression level and the X-axis represents different time points. The white and black bars below the graph represent day and night, respectively. Bamboo tissues were collected every 3 h over a 48-h period from one-month-old bamboo seedlings. The expression of genes at each time point was averaged using two days of data. *TIP41* was used to normalize the expression data. **a** Relative expressions of E2F group genes under abiotic stresses. **b** Relative expressions of DP group genes under abiotic stresses. **c** Relative expressions of DEL group genes under abiotic stresses

To understand the transcriptional changes in *PheE2F/DPs* during diurnal cycles, we investigated the expression profiles of the bamboo shoots at 4 h intervals over a 24 h period (Fig. 10). The qRT-PCR analysis indicated that most *PheE2FDPs* were regulated by diurnal cycles. The transcription levels of *PH02Gene07950.t1*, *PH02Gene31148.t3*, *PH02Gene03595.t1*, and *PH02Gene26414.t1* peaked at 15:00, 09:00, 06:00, and 12:00, respectively. The transcriptional levels of *PH02Gene34520.t1*, *PH02Gene18408.t2* and *PH02Gene42115.t1* remained at their maximum values from 4:00 to 19:00 (daytime).

## Discussion

### Features of the Moso bamboo E2F/DP gene family

E2F/DP controls the spatial and temporal expression of genes that are essential for multiple biological processes during the cell cycle, and their transcription level determined the cell proliferation [7]. In a genome-wide screening, 23 E2F/DP genes harboring conserved domains were identified in Moso bamboo, which was much higher than the numbers identified in rice (8), *Arabidopsis* (7), *Zea mays* (19), wheat (18) and *Brachypodium* (11) [15–17]. The Moso bamboo genome size (2021 Mb) is comparable to that of *Zea mays* (2300 Mb) [18], much smaller than that of wheat (*Triticeae*) (17 Gb) [19], and much larger than that of *Brachypodium* (300 Mb), *Arabidopsis* (164 Mb) and *Oryza sativa* (441 Mb) [20–22]. Rather than the genome size, the much higher number of *PheE2F/DPs* in Moso bamboo indicated that the abundance of *PheE2F/DP*s may be associated with genome duplication events*.* Previous reports suggested that Moso bamboo was tetraploid in origin, it went through a long evolutionary progression from tetraploidy to diploidy, and it carried two duplicates, similar to *Oryza sativa* gene model sets [13, 23]. A similar phenomenon was consistently observed in the E2F/DP family. Eight PheE2F/DP paralogous gene pairs located in synteny blocks were identified in the Moso bamboo genome. In addition, most divergence times of the PheE2F/DP paralogous pairs in Moso bamboo occurred 7–12 mya, consistent with the recent whole-genome duplication time. These results suggested that the whole-genome duplication event was a major driving force of PheE2F/DP family expansion.

### PheE2F/DP proteins are involved in Moso bamboo shoot development

Recently, several studies suggested that PheE2F/DP participated in many important biological processes in plants, such as leaf growth [24, 25], root growth [8], the maintenance of genome integrity and viability [26], and environmental stress response [27]. The expression analysis showed that more than half of the *PheE2F/DPs,* including five *E2Fs* and four *DPs,* showed the highest expression in winter bamboo shoots (Fig. 5). The screening of the binding sites at the promoter regions suggested that a large number of genes that participated in the cell cycle were regulated by the PheE2F/DP family, such as *CDKF*, *MRB1*, *SPR2* and *MCM2* (Fig. 6) [28], and most of these downstream genes also showed high expression levels during the winter and the early growth period. Previous studies suggested that intercalary meristem cells grew and divided continuously and vigorously during the winter and early growth periods (S1-S5), especially during the winter, but the intercalary meristem growth activity decreased during the late period, and the continuous growth of the bamboo shoot was substituted by cell elongation [2]. In *Arabidopsis*, E2Fa and E2Fb interact with DPa to regulate downstream gene expression [8]. In this study, we used the Y2H assay to detect the interaction between E2Fs and DPs, and three hub genes which showed the highest accumulation levels in winter bamboo shoots were selected and further examined. The experiment results showed that the E2F-DP complex also occurs in Moso bamboo, which provides favorable evidence that the PheE2F-PheDP complex may play important roles in winter bamboo shoot growth. Thus, we concluded that the high abundance of E2F/DP genes during the winter and early growth period was essential for E2F-DP complex formation and that the E2F-DP complex vigorously promoted meristem cell growth by activating the expression of the cell cycle genes.

A coexpression network analysis suggested that *PH02Gene2641.t1*, *PH02Gene05248.t2* and *PH02Gene01086.t1* were regulated by PH02Gene08546.t1 (MYB), PH02Gene20517.t1 (MYB) and PH02Gene25898.t2 (MYB). In addition, several MYB genes, such as *PH02Gene26882.t1* and *PH02Gene09101.t1,* repressed the expression of *E2F/DP* genes (Fig. 6). In tobacco, the MybA1 and MybA2 mRNAs fluctuated and peaked at the mitotic period and regulated the expression of the cell cycle genes. In the transient expression assays, MybA1 and MybA2 activated the MSA-containing (M-specific activator) promoters, whereas MybB repressed these promoters [29]. In the current study, all 23 promoters contained MYB binding sites, and most *E2F/DP* genes showed positive or negative correlations with *MYBs.* Thus, under the control of the MYBs, the *E2F/DP* genes played critical roles in regulating the expression of the cell cycle genes during Moso bamboo shoot growth and development.

### PheE2F/DP proteins are involved in stress and light responses

Based on the regulatory element analysis, a large number of light response sites were identified in the PheE2F/DP promoters. The expression analysis based on qRT-PCR also confirmed that most *PheE2F/DPs* were photoresponse genes. The transcriptional levels of *PH02Gene18408.t2* and *PH02Gene42115.t1* remained at the maximum value during the daytime in the Moso bamboo shoots, while *PH02Gene30693.t2* and *PH02Gene07670.t2* were highly expressed at night (Fig. 10). In *Arabidopsis*, light alters the balance of E2FC and E2FB, with opposing functions in the regulation of cell proliferation. Light increases the expression of E2FB protein levels and further induces the expression of cell cycle genes [30]. Thus, we speculated that *PH02Gene30693.t2* and *PH02Gene07670.t2,* which showed high accumulation levels at night, might act as negative regulators of the cell cycle and cell proliferation, and their high abundance at night might repress the expression of cell cycle genes. During the daytime, light increased the abundance of *PH02Gene18408.t2* and *PH02Gene42115.t1* and activated their expression, further accelerating light-mediated Moso bamboo shoot growth and development.

The identification of regulatory elements in the *PheE2F/DP* promoters indicated that most members harbored abscisic acid, MeJA and salicylic acid regulatory elements, indicating that the expression of these genes might be stimulated by some environmental cues. The qRT-PCR analysis also provided evidence that several *PheE2F/DP* genes were induced by abiotic stress (Fig. 9). Coexpression network analysis suggested that many environmental stress response genes were regulated by E2F/DPs. *PH02Gene44788.t1* (*AtRGGA*), which showed a high correlation with *PH02Gene41928.t1* (*E2F/DP*) and PH02Gene26414.t1 (*E2F/DP*), is involved in stress responsiveness. In *Arabidopsis*, *AtRGGA* is induced by salt and osmotic stress, and *atrgga* mutants exhibit increased sensitivity to abiotic stress [31]. In addition, *PH02Gene25752.t1* (an ABA-responsive gene) and *PH02Gene37465.t1* (a plant viral response gene) were also regulated by E2F/DPs in the network [32]. The high accumulation level of several *PheE2F/DPs* under abiotic stress might be essential for plant resistance to numerous adverse environments.

The regulation of the cell cycle might be subject to interference by drought and salt stress at the transcriptional level through repression of *E2F* expression. However, there is a great deal of new evidence that suggests that drought and salt stress can stimulate the expression of cell cycle genes. For the *Medicago truncatula E2Fb* gene, an upregulation trend was reported under high salt concentration treatment [3, 27]. In addition, many studies have also revealed the relationship between the accumulation level of cell cycle regulators and salt stress. The expression of *Oryza CDKC1,* which is involved in cell proliferation and differentiation, was triggered by NaCl treatment through the ABA signaling pathway [33]. In *Arabidopsis*, *CDC2aAt*, a cyclin-dependent kinase-coding gene and two mitotic cyclin genes, namely, *Arath;CycB1;1* and *Arath;CycA2;1,* were monitored during salt stress [34]. In mammals, many E2F members act as key factors in the DNA damage-dependent expression regulation of cell cycle genes [35]. Many PheE2F/DPs showed high accumulation levels under abiotic stress, which may facilitate bamboo growth under harsh environments. Collectively, *PheE2F***/***DPs* participated in different biological processes in Moso bamboo.

## Conclusion

In conclusion, a total of 23 PheE2F/DPs were identified in the Moso bamboo genome, including nine E2F, six DP, and eight DEL genes. The *cis*-acting elements involved in the photoperiodic response, hormone signaling, meristem growth, and many MYB and MYC2 binding sites were present in the *PheE2F/DP* promoters. Recent whole-genome duplication played important roles in the E2F/DP family expansion. Transcriptome sequencing and in situ hybridization analysis revealed that *E2F/DPs* play important roles in bamboo shoot growth, especially during the winter period. Expression profiles derived from qRT-PCR indicated that *E2F/DP* expression is stimulated by various forms of abiotic stress and diurnal cycles. These results provide comprehensive insights into the Moso bamboo E2F/DP gene family and lay a solid foundation for further functional verification of the PheE2F/DP family.

## Methods

### Database searches

The *E2F/DP* sequences of *Arabidopsis thaliana* (*Arabidopsis*), *Oryza sativa* (rice), and *Brachypodium distachyon* (*Brachypodium*) were downloaded from The Arabidopsis Information Resource (TAIR) [36], the rice genome annotation project [37], and the *Brachypodium distachyon* genome database [38], respectively. The published *AtE2F/DP* and *OsE2F/DP* sequences were then used as queries in BLASTP searches against the local bamboo protein database using the default parameters. The sequences were considered as predicted E2F/DPs if they had E-values of ≤ − 10. Lastly, the protein families database (Pfam) was further used to confirm each candidate E2F/DP sequence.

### Plant materials

Moso bamboo shoots as well as other culm samples were collected by Long Li in Guangde County (E119°41′; N30°89′), Anhui Province, from January to August 2018 (Additional file 1: Fig. S2). The formal identification of these culm samples were performed by Li et al. [2]. The bamboo samples were frozen in liquid nitrogen and stored at − 80 °C prior to RNA extraction. Permission to collect bamboo shoots and other culm samples for the experiments was obtained from the forestry bureau of Guangde County. Moso bamboo seeds were collected by Long Li in Dajing County, Guilin (E110°179′; N25°049′) in Guangxi Zhuang Autonomous Region from July to September, 2018 (Additional file 1: Fig. S2). The necessary field work permits were obtained from the Guilin Forestry Bureau. The formal identification of these seeds was performed by Gao et al. [39]. In addition, the sample collection work did not affect the ecology and did not involve protected species.

The circadian rhythm expression experiment was conducted in artificial climate chambers with supplemental light from 4:00 to 19:00. Bamboo tissues were collected by Long Li every 3 h during a 48-h period from one-month-old bamboo seedlings. Five individuals that showed similar growth patterns were pooled at each time point and three biological repeats were conducted for each sample.

For the abiotic stress treatment, bamboo seedlings were grown in artificial climate chambers under long-day conditions (15 h of light/9 h of dark). A drought stress test was conducted by irrigating the two-month-old seedlings with 18% PEG6000 medium. A salt stress test was conducted by irrigating the two-month-old seedlings with medium containing 250 mM NaCl. The leaves were harvested by Long Li at 1, 3, 12 and 24 h after abiotic stress treatment. Seedlings irrigated with tap water were used as the control (CK). The plant samples were frozen in liquid nitrogen and stored at − 80 °C prior to use. Five individuals that showed similar growth patterns were pooled at each time point and three biological repeats were employed for each sample. The voucher specimens were deposited in the herbarium at the International Center for Bamboo and Rattan, Beijing, catalog numbers ICBR-B-18101 (bamboo shoot), ICBR-B-18201 (rhizome), ICBR-B-18301(outward-rhizome), ICBR-B-18401 (seedling stem), ICBR-B-18501 (seed), ICBR-B-18601 (seedling leaf).

### Gene structure, conserved motif and regulatory element analysis

The Gene Structure Display Server program (GSDS) was used to investigate the gene structure of E2F/DP based on the GFF annotation file with the default parameters [40]. The conserved motifs in the identified E2F/DP sequences were identified using the Multiple EM for Motif Elicitation program (MEME) [41]. The MEME was run locally using the following parameters: number of repetitions - any, maximum number of motifs - 8, and the optimum motif widths were constrained to between 25 and 200 residues.

The transcription start sites (mRNA start sites) were designated as + 1 and obtained by GFF file as downloaded from the GigaDB, the new version of the Moso bamboo genome annotation project [23]. The regulatory elements in the promoter region of *PheE2F/DPs* as well as the other 51,050 Moso bamboo genes (from − 2000 bp to + 1 bp) were analyzed using the PlantCARE online program [14, 42]. A GO annotation of all the Moso bamboo genes was performed using TBtools based on the BLASTX method. BLASTX results with an e-value cutoff of 1e^− 6^ and a minimum similarity of 55% were annotated with GO terms. The promoters of genes that contained E2F/DP binding sites were further used for Gene Ontology (GO) enrichment analysis via TBtools [43].

### Collinearity analysis

To investigate the synteny of blocks containing *E2F/DP* genes, homology data derived from protein-protein comparisons made using BLASTN, with an E-value cutoff of 1e-6, and the other parameters were the defaults or recommended ones. The BLASTP result was analyzed using MCScanX to find the collinear regions between *PheE2F/DP* genes as well as other synteny blocks across the whole genome. The minimum block size was 5 and the other parameters were the default or recommended ones. The synteny blocks were extracted to draw a collinearity map within PheE2F/DPs using TBtools software.

### Estimation of the divergence time in paralogous pairs

The Ka (nonsynonymous substitution), Ks (synonymous substitution) and the Ka/Ks ratio between the paralogous pairs were calculated using DNA Sequence Polymorphism software (DnaSP). The formula T = Ks/2λ was used to estimate the divergence time of the duplication event, with a divergence rate λ = 6.5 × 10^− 9^ in monocotyledons and 1.5 × 10^− 8^ in dicotyledons [44].

### Multiple sequence alignment and phylogenetic tree construction

The ClustalW program was executed for multiple sequence alignments using the full-length E2F/DP amino acids with the default settings. The parameters used to run the CLUSTALW program were as follows: Scoring matrix for amino acid sequences: BLOSUM62, gap open: 10, gap extension: 0.2, iteration: none. The Maximum Likelihood tree was built using the IQ-Tree software with the best fit tree model, and the JTT + R3 was found to be the best model according to the Bayesian Information Criterion. The bootstrap method was applied in phylogeny test with 1000 replications. After that, the results were imported to FigTree v1.4.2 software for processing.

### RNA extraction, reverse transcription and qRT-PCR analysis

The total RNA from each sample was isolated using the TRIzol reagent (Invitrogen, USA). First-strand cDNA was synthesized with 2 μg RNA using a PrimeScriptTM RT Reagent Kit (TaKaRa, Japan) according to the manufacturer’s instructions. qRT-PCR experiments were performed using SYBR Green chemistry (Roche, Mannheim, Germany) on a Light Cycler 480 instrument (Roche, Rotreuz, Switzerland) according to the manufacturer’s directions. The Primer 3 online program was used for gene-specific primer design (Additional file 1: Table S2) [45]. Three biological replicates and three technical replicates were employed in each experiment. The TIP41 was selected as an internal control [46]. The final relative expression levels were calculated using the 2^-△△^Ct method.

### Transcriptome data analysis

The transcriptome data for *PheE2F/DPs* from the developing rhizome-root systems and bamboo shoots were previously generated and processed (PRJNA354950, PRJNA604634, PRJNA706151) [2, 12]. These transcriptome data were generated from different types of growing culms, including a 1.5-cm-tall seedling stem (SS1), 8-cm-tall seedling stem (SS2), lateral bud (L), rhizome (R), outward rhizome (O), and bamboo shoots at different growth stages (winter bamboo shoots and 50-, 100-, 300-, 600-, 900- and 1200-cm-tall shoots, designated S1-S7, respectively (Additional file 1: Fig. S2). Following leaf expansion, the culms were labeled as CK. The four growing culms (S1, O, SS1 and R) were sampled when the culm length reached one-tenth of the final length. The expression abundance of *PheE2F/DPs* was measured as fragments per kilobase of exon model per million mapped reads (FPKM). The heatmap was generated using R.

### Regulation network of PheE2Fs

The transcription factor-target gene (TF-TG) interactions were predicted based on the regulatory elements in the promoters of the target genes. The gene expression correlation was calculated by Pearson correlation coefficient (PCC) using FPKM through the R program. Only the transcription factor (TF) and target relationships that displayed PCC values of ≥ + 0.90 or < − 0.90 were retained for coexpression network construction. The network was ultimately visualized using Cytoscape 3.7.0 software.

### In situ hybridization

Bamboo shoot tissues were fixed in 4% paraformaldehyde for one day at 4 °C and then hybridized as described previously [2]. The specific probes of *PH02Gene01086.t1* and *PH02Gene26414.t1* were amplified by PCR using gene-specific primers with T7 and SP6 RNA polymerase-binding sites. The images were captured using an Olympus Nikon E600.

### Yeast two-hybrid assay

To test whether E2F can interact with DP in Moso bamboo, the Matchmaker GAL4 two-hybrid system (Clontech, Palo Alto, CA) was applied. Based on the in-fusion cloning method (Clontech, Palo Alto, CA), the full length of the PH02Gene26414.t1 and PH02Gene34005.t1 cDNAs were independently cloned into pGBKT7, and the full length PH02Gene34520.t1 cDNAs were cloned into pGADT7 (Additional file 1: Table S3). Next, the combinations of pGBKT7-PH02Gene26414.t1 + pGADT7-PH02Gene34520.t1 and pGBKT7-PH02Gene34005.t1 + pGADT7-PH02Gene34520.t1 were co-transformed into the AH109 yeast strain. The transformed yeasts were cultured on SD/−Trp/−Leu for 4 days and then transferred to SD/−Trp/−Leu/−Ade/−His/X-α-Gal plates for 5 days. The co-transformants containing pGBKT7–53 + pGADT7-T and pGBKT7-Lam + pGADT7-T were selected as the positive control and negative control, respectively.

## Supplementary Information

**Additional file 1: Figure S1**. Sequence logo of the different motifs identified in the PheE2F proteins. **Figure S2**. Moso bamboo tissues used for expression analysis. **Table S1**. Estimated divergence period of *E2F/DP* gene pairs in four species. **Table S2**. The primer sequences used for qRT-PCR. **Table S3**. The primer sequences used for gene cloning

## Data Availability

All the RNA-Seq raw data are available at NCBI under accession number PRJNA354950 (https://www.ncbi.nlm.nih.gov/bioproject/?term=PRJNA354950), PRJNA604634 (https://www.ncbi.nlm.nih.gov/bioproject/?term=prjna604634), PRJNA706151 (https://www.ncbi.nlm.nih.gov/bioproject/?term=PRJNA706151).

## Abbreviations

ABA: Abscisic acid
ABRE: Abscisic acid responsiveness
CDC: Cell division cycle
CDKC: Cyclin dependent kinase C
CREs: Cis-regulatory elements
DNnaSP: DNA Sequence Polymorphism
FPKM: Fragments per kilobase of exon model per million mapped reads
GSDS: Gene Structure Display Server program
GO: Gene ontology
Ka: Nonsynonymous substitution
Ks: Synonymous substitution
MEGA: Molecular evolution genetics analysis
MeJA: Methyl jasmonate
MEME: Multiple em for motif elicitation
MYA: Million years ago
NJ: Neighbor-joining
PCC: Pearson correlation coefficient
Pfam: Protein families database
PEG: Polyethylene glycol
qRT-PCR: Quantitative real-time PCR
TAIR: Arabidopsis Information Resource
TF-TG: Transcription factor-target gene
Y2H: Yeast two hybrid
