# Supplementary material for: Genome-wide identification and functional characterization of the PheE2F/DP gene family in Moso bamboo
Source: BMC Plant Biol. 2021 Mar 29;21:158. doi: 10.1186/s12870-021-02924-8 (PMC8008544; doi:10.1186/s12870-021-02924-8)
Supplement: Supplementary file 1 — Additional file 1: Figure S1. Sequence logo of the different motifs identified in the PheE2F proteins. Figure S2. Moso bamboo tissues used for expression analysis. Table S1. Estimated divergence period of E2F/DP gene pairs in four species. Table S2. The primer sequences used for qRT-PCR. Table S3. The primer sequences used for gene cloning [file 12870_2021_2924_MOESM1_ESM.docx]

Figure S1. Sequence logo of the different motifs identified in the *PheE2F* proteins


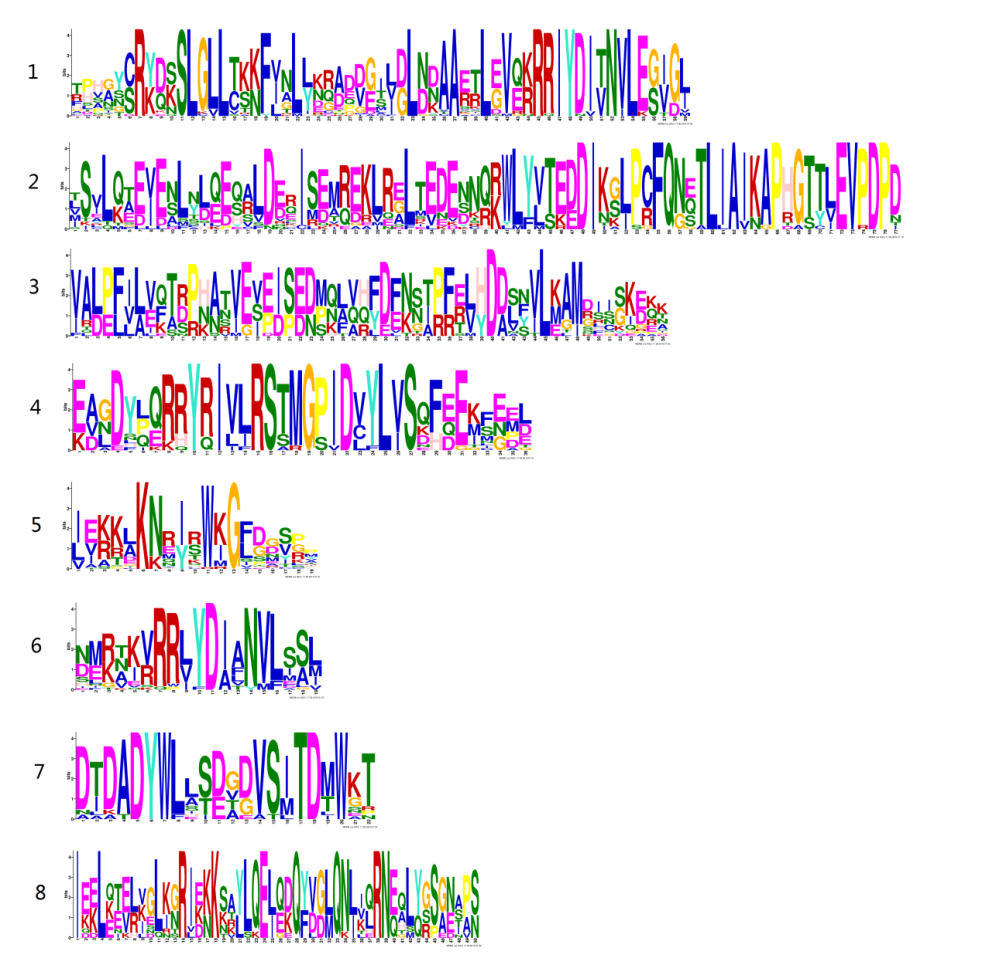


Figure S2. Moso bamboo tissues used for expression analysis. (A) moso bamboo shoot tips in seven growth stages (B) lateral bud, seedling stem and lateral bud samples, (C) seeds and seedlings used for experiment of circadian rhythms and abiotic stress treatment.


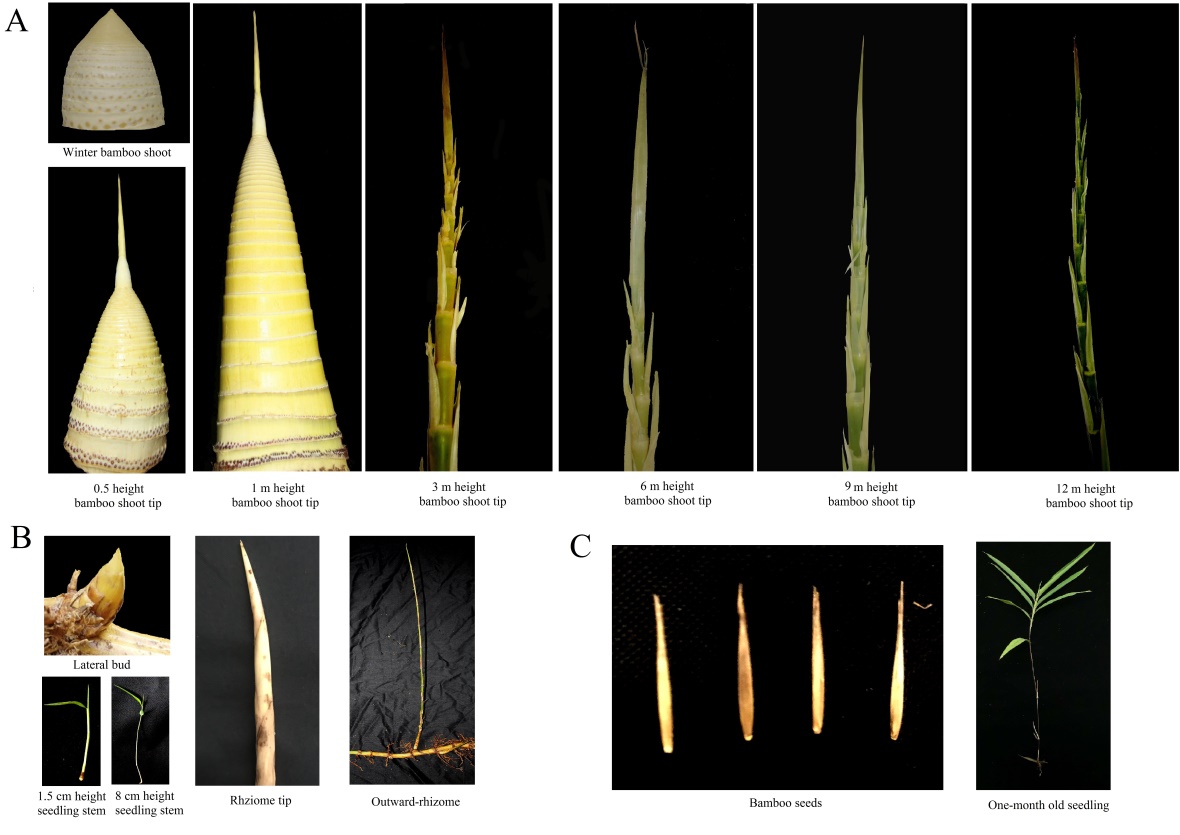


Table S1. Estimated divergence period of E2F/DP gene pairs in four species.

|  | ks | ka | ka/ks | Data (million years ago) |
| --- | --- | --- | --- | --- |
| PH02Gene41928.t1/PH02Gene30693.t2 | 0.1389 | 0.0366 | 0.263499 | 10.68 |
| PH02Gene43494.t1/PH02Gene07670.t2 | 0.0909 | 0.0358 | 0.393839 | 6.99 |
| PH02Gene34520.t1/PH02Gene43370.t2 | 0.1474 | 0.0371 | 0.251696 | 11.33 |
| PH02Gene09693.t3/PH02Gene31148.t1 | 0.1337 | 0.0472 | 0.353029 | 10.28 |
| PH02Gene39656.t1/PH02Gene26414.t1 | 0.1025 | 0.0319 | 0.31122 | 7.88 |
| PH02Gene03595.t1/PH02Gene10996.t1 | 0.1741 | 0.0987 | 0.566916 | 13.39 |
| PH02Gene34005.t1/PH02Gene20868.t1 | 0.0931 | 0.0463 | 0.497315 | 7.15 |
| PH02Gene01086.t1/PH02Gene05248.t2 | 0.7562 | 0.1525 | 0.201666 | 70.27 |
| AT2G36010.1/AT1G47870.1 | 1.2146 | 0.6697 | 0.551375 | 40.49 |
| AT5G02470.1/AT5G03415.1 | 0.4962 | 0.6477 | 1.30532 | 16.54 |
| AT5G14960.1/AT3G01330.1 | 0.5613 | 0.2787 | 0.496526 | 18.71 |
| Bradi3g45130.1/Bradi5g09640.1 | 0.7207 | 0.2778 | 0.385459 | 55.44 |
| Bradi4g42050.1/Bradi5g01920.1 | 0.6292 | 0.6507 | 1.03417 | 48.4 |
| Bradi1g74890.1/Bradi3g27520.1 | 0.7789 | 0.1864 | 0.239312 | 59.91 |
| Bradi1g44520.1/Bradi3g59740.1 | 0.4232 | 0.4087 | 0.965737 | 32.55 |
| LOC_Os02g33430.1/LOC_Os04g33950.1 | 0.2713 | 0.3032 | 1.117582 | 20.87 |
| LOC_Os12g06200.1/LOC_Os04g02140.1 | 0.7619 | 0.6714 | 0.881218 | 58.61 |
| LOC_Os01g48700.1/LOC_Os03g05760.1 | 1.5446 | 0.9857 | 0.638159 | 11.88 |
| LOC_Os06g13670.1/LOC_Os02g50630.1 | 0.872 | 0.1912 | 0.219266 | 67.07 |

Table S2. The primer sequences used for qRT-PCR.

| Primer names | Primers |
| --- | --- |
| PH02Gene01086.t1F | CAAGAGAGCCTTTGGGACTG |
| PH02Gene01086.t1R | GAGCTCCTGTTTCCTTGCAC |
| PH02Gene03595.t1F | AGAGCTCAAGCATGCCAAAT |
| PH02Gene03595.t1R | TCAAGATCGACCCATCACAA |
| PH02Gene05248.t2F | GCAAGCGGACTCAAGAAAAC |
| PH02Gene05248.t2R | TGGCTTTGTTCTCTGTCGTG |
| PH02Gene07670.t2F | GCTACCCCTGACGACTACCA |
| PH02Gene07670.t2R | GCAGCATCACTTTCTCCACA |
| PH02Gene07950.t1F | CAGATGACGGAACCCTTGAT |
| PH02Gene07950.t1R | GTTTGTTCGTGCCCTTCAAT |
| PH02Gene09693.t3F | AGAGAAACTGCAAGCCCTCA |
| PH02Gene09693.t3R | GGGACCCATTGAACTTCTCA |
| PH02Gene10996.t1F | GCAGATGAACTTGTCGCTGA |
| PH02Gene10996.t1R | CCTACCTTTCAGTCCCACGA |
| PH02Gene18408.t2F | CTCCAAAGCTGGTGAAGGAG |
| PH02Gene18408.t2R | TTGGTGGGGGTACAACAACT |
| PH02Gene20868.t1F | ATGCAGTTGGTGCATTTTGA |
| PH02Gene20868.t1R | GCAGGTTGTGGTGATTGATG |
| PH02Gene26414.t1F | TGGAGGAAGTTCGCAAAGAT |
| PH02Gene26414.t1R | GAAGGCGGATACCATTAGCA |
| PH02Gene30693.t2F | GGGGTGGTGGTGATATTGAG |
| PH02Gene30693.t2R | CAACATTTGTCGTCGGTGTC |
| PH02Gene31148.t1F | CTGCAGCAGGTGGATTATGA |
| PH02Gene31148.t1R | AGCGAGCCAGTAATCAGCAT |
| PH02Gene34005.t1F | TGGTACAGCGAAATGAGCAG |
| PH02Gene34005.t1R | GAGAACCCCATTGCTTTCAA |
| PH02Gene34267.t1F | ACGTGGAGTCTATCGGGTTG |
| PH02Gene34267.t1R | CTCTCAATGCCCTCTCCTTG |
| PH02Gene34520.t1F | GCACAGCTTCGATGAACAAA |
| PH02Gene34520.t1R | TCATCAGGATCTGGGACCTC |
| PH02Gene42115.t1F | GGGCTGTAGTTGTGGCAGTT |
| PH02Gene42115.t1R | TCTCCAAGGAGCAGCTTTGT |
| PH02Gene43370.t2F | TGCACAGATGGAACCGAATA |
| PH02Gene43370.t2R | CCTCTGAGGCTGTCTTCCAC |
| PH02Gene43494.t1F | AGGACGCCCCTAAAAAGAAA |
| PH02Gene43494.t1R | AGTAGGTGGATTGCCAGGTG |
| TIP41F | AAAATCATTGTAGGCCATTGTCG |
| TIP41R | ACTAAATTAAGCCAGCGGGAGTG |

Table S3. The primer sequences used for gene cloning.

| Primer names | | Primers | |
| --- | --- | --- | --- |
| PH02Gene26414.t1F | | **GGCCGAATTCCCGGGG** ATGCATGCTCCTCTCCAGTCT | |
| PH02Gene26414.t1R | | **GCCGCTGCAGGTCGAC** TCAATGAAGGGTGCCCAGG | |
| PH02Gene34005.t1F | | **GGCCGAATTCCCGGGG** ATGGTCTCCGGCACCCGTA | |
| PH02Gene34005.t1R | | **GCCGCTGCAGGTCGAC**TCAATGTTCATGCTTGACACGC | |
| PH02Gene34520.t1F | | **GCAGAGTGGCCATTATGGCCC**ATGGCGGCGACCGGCGGT | |
| PH02Gene34520.t1R | | **GCGGCCGACATGTTTTTTCCC**TCAGCTTATGCAGGTGGGGA | |
